# Exploring Smartphone Use Among Secondary School Students in a Rural School in Bangladesh: A Cross‐Sectional Pilot Study

**DOI:** 10.1002/puh2.70158

**Published:** 2025-10-24

**Authors:** Salma Afroz, Ali Saifullah Sajib, Faysal Ahmed, Arjun Kumar Ghosh, Masood‐Ur Rahman

**Affiliations:** ^1^ Pediatric Neurology National Institute of Neuroscience Dhaka Bangladesh; ^2^ Directorate General of Health Services Dhaka Bangladesh; ^3^ Cardiac Surgery National Institute of Cardiovascular Diseases Dhaka Bangladesh; ^4^ National Institute of Cardiovascular Diseases Dhaka Bangladesh; ^5^ Gastroenterology Dhaka Medical College and Hospital Dhaka Bangladesh

**Keywords:** adolescent, Bangladesh, smartphone

## Abstract

**Background:**

Smartphone use is increasingly common among adolescents, but its impact on academic performance, health, and well‐being in rural Bangladesh is poorly understood. This study explored usage patterns, purposes, and potential effects among rural adolescents.

**Aim:**

To explore smartphone usage patterns, purposes of use, and associated health and lifestyle effects among secondary school students in a rural region of Bangladesh.

**Methods:**

This cross‐sectional study was conducted among secondary school students in a village of Chandpur district. Data were collected using a self‐reported structured questionnaire. Statistical analysis was performed using SPSS version 22.

**Results:**

Among the participants, 60% were male and 40% female; most were in Grade 10 (33.33%). Daily smartphone use was high, with 43.33% using smartphones for 3–5 h and 21.67% for over 5 h. Entertainment (45%) and communication (22%) were primary uses, whereas only 18.33% used smartphones for study. Facebook (61.67%) and TikTok (56.67%) were most popular. Educational app use was limited, with 81.67% not using any academic apps; YouTube and Zoom were most common among users. Over half (55%) engaged in extra non‐academic activities, including online gaming (61.67%). Outdoor activity was limited, and 65% reported being scolded for smartphone use. Common health complaints included decreased concentration (63.33%), headaches (41.67%), vision problems (38.33%), poor academic performance (33.33%), decreased sleep (23.33%), and back pain (21.67%).

**Conclusion:**

Smartphone use is widespread among rural Bangladeshi adolescents, mainly for entertainment and communication, with limited academic engagement. High usage is associated with concentration and health issues. Larger studies and interventions promoting digital literacy and healthy smartphone habits are needed to optimize benefits and minimize risks.

## 1 Background

Smartphones have rapidly become central to adolescents’ lives worldwide, influencing how they communicate, learn, and entertain themselves. Today, almost 95% of adolescents worldwide own or have access to a smartphone, reflecting a sharp increase in ownership over the past decade [1, 2]. In Bangladesh, where adolescents comprise almost 22% of the population, smartphone penetration is increasing in parallel with expanding internet coverage and affordability. A recent survey indicates that over 90% of Bangladeshi adolescents use smartphones, highlighting their growing dependence on digital technologies [3]. Although this digital expansion has the potential to improve educational access, particularly in rural areas where traditional resources are scarce, it also raises concerns about academic distraction, reduced physical activity, and adverse health effects [4, 5].

In rural areas, where access to conventional educational resources remains limited, smartphones create new opportunities for mobile learning [6]. They enable access to e‐books, video lectures, and interactive applications that can enrich the educational experience and support students beyond the classroom [4, 7]. Evidence from different settings suggests that mobile‐assisted learning can improve academic performance, digital literacy, and student engagement [8]. Such adoption is often explained through the uses and gratifications theory, which highlights that adolescents choose digital media to satisfy needs for information, entertainment, and social interaction [9]. On the other hand, unregulated use is increasingly associated with harmful consequences. Studies link excessive smartphone use to shorter attention spans, procrastination, poor sleep quality, and higher levels of anxiety and depression [10]. Studies suggest that the time devoted to smartphones replaces important offline activities, such as studying, exercise, and family interaction [11]. Health complaints, such as headaches, eye strain, musculoskeletal discomfort, and sleep disturbance are also frequently reported among adolescents with high screen exposure [12]. Taken together, these findings show that smartphones may act as both teaching tools and health risks depending on usage patterns.

Although global literature has examined adolescent smartphone use extensively, important contextual gaps remain. Most studies from Bangladesh have focused on urban populations or university students, with limited exploration of rural adolescents who often face unique socio‐cultural constraints, lower digital literacy, and weaker parental supervision [13, 14]. In particular, little is known about how rural Bangladeshi adolescents balance educational and recreational use or how these patterns influence health and lifestyle. Against this backdrop, the present study addresses a critical gap in the literature. The problem lies in the lack of empirical evidence about smartphone use among rural secondary school students in Bangladesh, despite the rapid digital expansion in these settings. This gap limits the capacity of educators, parents, and policymakers to design appropriate interventions that maximize educational benefits while minimizing risks. The guiding research question for the current study is: *What are the patterns, purposes, and health consequences of smartphone use among rural secondary school students in Bangladesh?* So this study aims to investigate smartphone usage patterns among secondary school students in rural Bangladesh, focusing on their educational engagement, patterns of misuse, and the resulting effects on their physical health. This study is significant because it provides a baseline evidence that will inform awareness campaigns, parental guidance strategies, and school‐based interventions promoting balanced smartphone use.

## 2 Methods

### 2.1 Study Design and Setting

This descriptive cross‐sectional study was conducted at a secondary school in Chandpur, Bangladesh, over a 3‐day period (March 27–29, 2025). Data collection was performed on‐site within classrooms during school hours.

### 2.2 Study Population and Eligibility

The target population comprised students enrolled in Grades 6–10.

**Inclusion criteria**

- Enrollment in Grades 6–10 at the selected school.
- Ownership or access to a smartphone (either personally owned or owned by a parent/guardian).
- Ability to understand and respond to the questionnaire.
- Willingness to participate, with written parental/guardian consent and student assent.

**Exclusion criteria**

- Students absent or on leave during the data collection period.
- Students or guardians who declined to provide written informed consent.
- Students with significant communication or cognitive difficulties preventing questionnaire completion.

### 2.3 Sample Size Calculation

Sample size was calculated using the following formula:

$$n=Z^{2}p\left(1-p\right)/d^{2}$$

where *n* is the required sample size, *Z* is 1.28 (for 80% confidence level), *p* is the estimated prevalence of 90% (based on usage of smartphones among Bangladeshi adolescents), and *d* is 0.05 (5% margin of error).

On the basis of this formula, the required sample size is approximately 59 participants.

### 2.4 Sampling Method

The sample was obtained through the census method based on the predefined inclusion and exclusion criteria. All students present at the school during the data collection period who met the eligibility criteria were invited to participate. The school had a total of 100 students, of whom 65 were present during the data collection period. Ultimately, 60 students met the inclusion criteria and provided informed consent, resulting in a final sample size of 60 participants.

### 2.5 Data Collection Procedure

Data were collected using a structured questionnaire assessing smartphone access, usage duration, purpose of use, outdoor activity, and health complaints. The questionnaire was developed in English, translated into Bangla, and back‐translated by bilingual experts. Three experts in public health and education reviewed the content for clarity and cultural appropriateness. A pilot test with 10 Grade V students at a neighboring primary school assessed comprehensibility and response burden. On the basis of feedback, wording was simplified. Internal consistency was confirmed (Cronbach's *α* = 0.8).

Data collection occurred in classrooms under the supervision of trained research assistants. The study purpose was explained to students, and participation was voluntary. To minimize selection bias, all eligible students present were invited to participate. To reduce information bias, the questionnaire was anonymous, questions were explained uniformly, and students completed it at their desks independently without interference from teachers or parents. Research assistants were present to provide clarifications in a neutral manner without leading participants. The classroom environment was structured to ensure privacy and minimize peer influence. Completed questionnaires were returned immediately.

To ensure data quality, completed questionnaires were checked immediately for missing responses. Questions regarding academic performance and decreased concentration were verified and corrected in consultation with the students’ teachers and guardians. Ten percent of questionnaires were randomly rechecked by the principal investigator for accuracy of data entry and coding.

### 2.6 Statistical Analysis

Collected data were coded, entered, and analyzed using SPSS version 22. Categorical variables are reported as frequencies and percentages and graphs. Continuous variables were presented as mean, median, and SD. Means were calculated with a 95% confidence interval, and *p* values <0.05 were considered significant. The study adhered to the *STROBE* checklist for cross‐sectional studies to ensure methodological transparency and completeness.

### 2.7 Ethical Considerations

The study protocol was reviewed and approved by the Ethical Review Committee (Letter No.: 05.05.0000.003.01.099.25.82; Date: 20/03/2025) of the BIAM Foundation, Dhaka, Bangladesh. Written informed consent was obtained from parents/guardians, and verbal assent was obtained from the students before administration of the questionnaire.

## 3 Results

Among the 60 participants, 60% were male and 40% female. Most were aged 15–17 years (68.33%) and enrolled in Grade 10 (33.33%), followed by Grade 9 (26.67%) and Grade 8 (21.67%) (Table 1).

**TABLE 1:** Socio‐demographic characteristics of the study participants (*n* = 60).

Variables	Frequency	Percentage (%)
**Gender**		
Male	35	60
Female	25	40
**Age**		
12–14	22	36.60
15–17	38	68.33
**Grade**		
Grade 6	4	6.67
Grade 7	7	11.67
Grade 8	13	21.67
Grade 9	16	26.67
Grade 10	20	33.33

Daily smartphone use was high, with 43.33% of participants using smartphones for 3–5 h and 21.67% for more than 5 h. Only 6.67% used them for less than 1 h. The primary purposes of use were entertainment (45%) and communication (22%), while only 18.33% used smartphones for study‐related activities. Facebook (61.67%) and TikTok (56.67%) were the most frequently used social media platforms (Table 2).

**TABLE 2:** Daily smartphone usage pattern among study participants (*n* = 60).

Variables	Frequency	Percentage (%)
**Duration**		
Less than 1 h	4	6.67
1–3 h	17	28.33
3–5 h	26	43.33
More than 5 h	13	21.67
**Purpose of use**		
Entertainment	27	45
Communication	27	22
Studying	11	18.33
**Use of social media by participants**		
Facebook	37	61.67
TikTok	34	56.67
WhatsApp	18	30
YouTube	21	35
Imo	15	25
Instagram	12	20
Snapchat	9	15

Educational use of smartphones was limited, as 81.67% of students did not use academic apps. Among those who did, YouTube (27.8%) and Zoom (27.8%) were the most common platforms. Over half of the participants (55%) spent extra time on non‐academic activities, with online gaming reported by 61.67% of students (Table 3).

**TABLE 3:** Academic and other uses of smartphone among study participants (*n* = 60).

Variables	Frequency	Percentage (%)
**Apps for online class**		
No	49	81.67
Yes	11	18.33
Zoom	3	27.8
YouTube	5	27.8
Ten Minute School	2	22.2
ChatGPT	1	5.6
**Extra time spent outside study**		
Yes	33	55
No	27	45
**Non‐academic use**		
Online gaming	37	61.67
Reels and meme watching	23	38.33

Most participants (65%) reported being scolded for smartphone use. Outdoor activity was limited, with 50% playing for less than 1 h and only 8.3% engaging in outdoor play for more than 3 h. Common health issues included decreased concentration (63.33%), headaches (41.67%), and blurring of vision (38.33%). Other reported problems were poor academic performance (33.33%), decreased sleep at night (23.33%), and back pain (21.67%) (Table 4).

**TABLE 4:** Social and health impacts of smartphone uses among study participants (*n* = 60).

Variables	Frequency	Percentage (%)
**Family and teacher opinion about smartphone use outside educational activities**		
Scolding	39	65
Don't say anything	21	35
**Playing outdoor**		
None	5	8.3
<1 h	30	50
1–3 h	20	33.3
>3 h	5	8.3
**Health impacts**		
Decreased concentration	38	63.33
Headache	25	41.67
Blurring of vision	23	38.33
Poor academic performance	20	33.33
Decreased sleep at night	14	23.33
Back pain	13	21.67

## 4 Discussion

This study explored patterns of smartphone use, its purposes, and associated health and lifestyle effects among rural secondary school students in Bangladesh. The findings show that smartphones are primarily used for entertainment and communication rather than education, with 43.3% of students spending 3–5 h per day on their devices and 21.7% reporting more than 5 h of use. This result is consistent with previous research; for example, Mondal et al. reported that around half of early adolescents in Bangladesh spend at least 3 h daily on smartphones or other digital devices [6]. Even in the United States, teenagers average more than 4 h of screen media use per day outside of schoolwork [15]. Prolonged screen time was associated with limited outdoor physical activity, with half of the students in our study engaging in less than 1 h of outdoor play per day. These findings support the displacement hypothesis, which suggests that time allocated to screen use replaces more beneficial activities such as physical exercise or study [16]. Similar displacement effects have been documented from previous studies from Bangladesh and internationally [3, 15, 17]. The extent of displacement may be amplified in rural Bangladesh, where fewer organized recreational opportunities exist. Unlike urban students who may participate in structured sports or extracurricular activities, rural adolescents often rely on unstructured outdoor play. Consequently, when recreational smartphone use dominates, physical activity can decline more sharply, exacerbating risks of obesity, poor sleep, and reduced social interaction [13]. A cohort study suggested that higher screen time in preadolescents and adolescents was linked to a greater likelihood of poor physical, emotional, and school‐related quality of life, as well as social difficulties in adolescents [11].

A key finding of our study was that only 18.3% of participants reported using smartphones for study purposes, whereas others engaged in social media, gaming, and video streaming. This reflects a mismatch between the educational potential of smartphones and their actual use by adolescents. Similar findings have been reported in previous Bangladeshi studies. Haq et al. observed that over 80% of secondary school students in Kotalipara primarily used smartphones for entertainment, and Islam found that even urban adolescents in Dhaka prioritized social networking over academic applications [13, 17]. At the international level, our findings are consistent with evidence from Europe, the Middle East, and North America, where adolescents also spend the majority of their screen time on entertainment, online gaming, and social networking rather than educational activities [18, 19]. However, contrasting evidence shows that when properly guided, mobile learning can enhance student engagement and academic outcomes. For instance, two separate studies demonstrated that mobile‐assisted learning significantly improved vocabulary acquisition and improved performance outcomes [8, 20]. The divergence between these positive outcomes and our findings highlights a missed opportunity in rural Bangladesh, where smartphones are present but insufficiently integrated into structured educational practices. This underutilization of digital platforms may be due to infrastructural challenges, lack of teacher training, and concerns over screen‐related health risks as major barriers to digital learning in rural Bangladesh [14].

We found that 63.3% of students reported reduced concentration, 41.7% experienced headaches, and 38.3% suffered vision problems due to overuse of smartphones. These findings are consistent with previous Bangladeshi studies, which similarly documented that excessive gadget use among children was associated with high rates of headaches and vision problems, as well as concentration difficulties and sleep disturbances among adolescents [3, 13]. A systematic review also suggests that excessive smartphone use is associated with depression, anxiety, and cognitive fatigue [21]. In line with this evidence, our study found similar adverse outcomes, as reflected in the high proportion of students reporting decreased concentration (63.3%) and poor academic performance (33.3%). In rural Bangladesh, where academic environments are already resource‐constrained, the added cognitive burden of prolonged recreational smartphone use may further undermine learning outcomes.

Another noteworthy result was that 65% of students had been reprimanded for smartphone use. This reflects parental and teacher concerns over excessive or inappropriate usage, consistent with findings from Munira and bin Ahsan, who reported widespread skepticism among Bangladeshi rural adults regarding the educational value of smartphones [14]. In contrast, parents often associate smartphone use with distraction rather than learning in urban areas [13]. Internationally, Livingstone and Blum‐Ross observed similar tensions, noting that while parents recognize potential benefits, they often resort to restriction rather than guided mediation [22]. In rural Bangladesh, where digital literacy among adults is comparatively low, parental strategies are more prohibitive than supportive, which may limit constructive smartphone use and increase generational conflict.

## 5 Limitations

This study has several limitations. The small sample size (*n* = 60) from a single, non‐randomly selected school limits generalizability and statistical power. The cross‐sectional design prevents conclusions about causality. The study lacks an in‐depth examination of relationships between smartphone use and academic or health outcomes, as well as psychological effects (anxiety, depression, cyberbullying) and socioeconomic factors (family income, parental education). Reasons for limited academic use of smartphones, such as infrastructure or teacher training, were not explored.

## 6 Conclusion

This study highlights prevalent smartphone use among rural Bangladeshi adolescents, predominantly for social and entertainment purposes, with limited academic engagement. Although smartphone use offers connectivity and recreational benefits, potential adverse effects on concentration, sleep, and psychological well‐being were observed. The findings underscore the need for larger, multi‐site studies to explore the complex relationships between smartphone use, academic performance, health outcomes, and socioeconomic factors.

## Author Contributions

**Salma Afroz:** conceptualization, methodology, data collection, supervision, writing and review; **Ali Saifullah Sajib:** methodology, data collection, data analysis, writing and review; **Faysal Ahmed:** conceptualization, data collection, writing and review; **Arjun Kumar Ghosh:** conceptualization, data collection, writing and review; **Masood‐Ur Rahman:** conceptualization, data collection, writing and review

## Conflicts of Interest

The authors declare no conflicts of interest.

## Data Availability

The data that support the findings of this study are available from the corresponding author upon reasonable request.
